# Numerical Study on Surface Roughness Measurement Based on Nonlinear Ultrasonics in Through-Transmission and Pulse-Echo Modes

**DOI:** 10.3390/ma14174855

**Published:** 2021-08-26

**Authors:** Maodan Yuan, Anbang Dai, Lin Liao, Yan Chen, Xuanrong Ji

**Affiliations:** State Key Laboratory of Precision Electronic Manufacturing Technology and Equipment, School of Electromechanical Engineering, Guangdong University of Technology, Guangzhou 510006, China; mdyuan@gdut.edu.cn (M.Y.); anbangdai@163.com (A.D.); L2630375128@163.com (L.L.); yanchen@gdut.edu.cn (Y.C.)

**Keywords:** nondestructive testing, surface roughness, nonlinear ultrasonic, through-transmission, pulse echo

## Abstract

Ultrasonic is one of the well-known methods for surface roughness measurement, but small roughness will only lead to a subtle variation of transmission or reflection. To explore sensitive techniques for surfaces with small roughness, nonlinear ultrasonic measurement in through-transmission and pulse-echo modes was proposed and studied based on an effective unit-cell finite element (FE) model. Higher harmonic generation in solids was realized by applying the Murnaghan hyperelastic material model. This FE model was verified by comparing the absolute value of the nonlinearity parameter with the analytical solution. Then, random surfaces with different roughness values ranging from 0 μm to 200 μm were repeatedly generated and studied in the two modes. The through-transmission mode is very suitable to measure the surfaces with roughness as small as 3% of the wavelength. The pulse-echo mode is sensitive and effective to measure the surface roughness ranging from 0.78% to 5.47% of the wavelength. This study offers a potential nondestructive testing and monitoring method for the interfaces or inner surfaces of the in-service structures.

## 1. Introduction

In practical engineering problems, the structure surface is always far from ideally flat and smooth and varies from specimen to specimen. Such an imperfect surface might affect the mechanical performance, including wear resistance, fatigue strength, and corrosion resistance of the components. Although various surface treatments such as polishing, lapping, and milling could be applied to reduce the surface roughness, it may not be easy to prepare perfect surfaces to the required level due to the high need for manpower, machinery, and economic cost. Moreover, the surface state of in-service structure probably changes with time. Therefore, as one of the key indicators of surface texture, it is of importance to evaluate surface roughness during the manufacturing and the service process.

Typical roughness measurement methods include stylus sensing, optical interferometry, and scanning tunneling microscopy. These profiling methods can provide detailed information of the surface topology. But abrasion and scratches might be introduced to the surface during stylus sensing and optical methods are applicable primarily to the roughness less than 1 µm due to the limitation of light wavelength. For the industrial components manufactured by common processes like milling, shot peening, and chemical deposition, the surface roughness ranges from about 0.5 µm to 50 µm. Moreover, most current methods are applicable only to accessible top surfaces. In a practical engineering application, the information of interfaces [1,2] or inner surfaces [3] are sometimes also demanded. To balance the resolution and penetration capability, ultrasonic method is the optimal choice. Different from the profiling methods, the ultrasonic method is a kind of averaging of methods, providing a representative parameter of the statistical properties of the rough surfaces in the investigated area.

The application of ultrasound to assess the surface roughness of industrial components began in 1980s. Kirchhoff approximation (KA), based on the Huygens’ principle, was applied to predict the wave field scattered from a rough surface [4]. This approximation is exact only for small-amplitude and small-slope surfaces, in which the correlation length $L_{c}$ is much larger than the root-mean-square (RMS) height $R_{q}$. In the low-frequency limit of KA, a phase-screen approximation (PSA) was derived to analyze the interaction between the ultrasonic wave and the rough surfaces [5,6,7]. Lian et al. [8] derived the scattering attenuation with the consideration of the influence of local incident angle. Experimental studies demonstrated that the measurement errors were less than 10% when the relationship between roughness $R_{q}$ and wavelength $\lambda_{w}$ satisfied $R_{q}=1.6\sim 10.0\%\;\lambda_{w}$ [2]. When the roughness $R_{q}$ is too large, the non-coherent wave field caused by the rough interface cannot be ignored and the measurement errors are large. Recently, Shi et al. proposed elastodynamic KA to predict the scattered and transmitted wavefield for a wider range of roughness up to $R_{q}\;\leq \;\lambda_{w}/3$ [9,10,11]. The proposed analytical theory has been applied to reconstruct the real rough surface and its correlation function [12,13,14]. On the other hand, a small roughness $R_{q}$ will lead to only a subtle variation of transmission or reflection, and thus it will not be able to accurately measure the small roughness by conventional ultrasonic methods. Therefore, a higher sensitive method is required.

A potential alternative is nonlinear ultrasonic methods based on the nonlinear response, which is able to track the onset of early-stage damage, such as material degradation [15,16] and closed cracks [17,18,19]. The surface roughness of a specimen has a negative but not neglected effect during the nonlinear ultrasonic measurement [15]. In the current laboratory-based experiment of the nonlinear ultrasonic testing, surface roughness should be removed as much as possible when preparing the specimens [15,17]. However, the components to be monitored in the industrial applications have random surfaces. Therefore, a detailed investigation of the effects of surface roughness is in great demand to provide guidance for the in-situ structural health monitoring (SHM) based on nonlinear ultrasonics. Na et al. made an initial attempt to investigate the variation of ultrasonic nonlinearity parameter with different surface roughness and nonparallelism [20]. PSA theory was adopted to explain the experimental results, but there were some mismatches, especially when the roughness became large. Chakarapani et al. experimentally studied the influence of surface roughness on nonlinearity parameter using contact piezoelectric transducers [21]. Recently, Kim et al. extended the analysis for the specimens with single and double rough surfaces [22].

However, these researches were all conducted in the through-transmission mode rather than the pulse-echo mode. The reason lies in that higher harmonic accumulated in forward propagation decreases to zero when it reflects back from the stress-free boundary to the source position [23]. This phenomenon makes it difficult to obtain reliable values for the nonlinearity parameter β in the pulse-echo mode. However, pulse-echo measurement is much more desirable for practical applications due to the single-side setup. Best et al. [24] detected nonlinearity with a dual element transducer in the pulse-echo mode, because the diffraction effects of the sound beam field were considered. Zhang et al. [25] employed a focused transducer to measure second harmonic reflected from a free surface. More recently, Jeong et al. [26,27] experimentally demonstrated the feasibility to measure the nonlinearity parameter β in the pulse-echo mode with an optimized dual-element transducer. To the best of the authors’ knowledge, there is no study on roughness measurement based on nonlinear ultrasonic, especially in the pulse-echo mode. Therefore, a comprehensive study should be carried out on the interaction between higher harmonics and rough surfaces in different measurement setups. To bridge the gap between the existing analytical predictions and experimental measurement for ultrasonic wave scattering from rough surface, numerical methods, such as finite element (FE) modelling, are widely applied [13,28,29]. These studies will demonstrate the feasibility of surface roughness measurement based on nonlinear ultrasonics. Besides, the study on ultrasonic wave scattering from rough surfaces is also beneficial for the enhancement of ultrasonic thickness measurements [3] and the detection of rough cracks or delamination in real cases [29,30,31].

In this study, we focus on the investigation of the feasibility of surface roughness measurement based on the nonlinear ultrasonic in both through-transmission and pulse-echo modes. First, the basic theory for ultrasonic higher harmonic generation based on the plane wave theory and ultrasonic scattering from rough surfaces will be briefly given in Section 2 and Section 3. An efficient FE model for nonlinear ultrasonic testing will be described in Section 4. Based on this verified FE model, the results will be discussed for surface roughness measurement in both through-transmission and pulse-echo modes. Finally, conclusions will be drawn in Section 6.

## 2. Higher Harmonic Generation in Solid Media

Numerous works had introduced the derivation and solution of the nonlinear ultrasonic wave equation. Following Green’s approach and notations [32], the differential equation of wave motion in a form with separate linear and nonlinear terms is written as Equation (1):

(1) $$\rho \frac{\partial^{2}u}{\partial t^{2}}=E_{1}\frac{\partial^{2}u}{\partial x^{2}}+E_{2}\frac{\partial u}{\partial x}\left(\frac{\partial^{2}u}{\partial x^{2}}\right)+\ldots =E\left\{\frac{\partial^{2}u}{\partial x^{2}}+\beta \frac{\partial u}{\partial x}\left(\frac{\partial^{2}u}{\partial x^{2}}\right)+\ldots \right\}$$

where ρ is the density; u is the particle displacement in the x-direction; $E_{1}=E$ and $E_{2}=\beta E_{1}$ give the expressions of elastic constants. It should be noted that $E_{1}$ is expressed in terms of second-order elastic constants only, while $E_{2}$ is expressed in terms of both second- and third-order elastic constants. Using the perturbation technique, the displacement solution of Equation (1) can be expressed as $u=u^{0}+u^{1}$, where $u^{0}$ is the linear response; $u^{1}$ is the perturbation term; and $u^{1}\ll u^{0}$. The trial solution, $u^{0}=A\sin\left(kx-\omega t\right)$ is used, where A is the incident displacement amplitude, and k and ω are the wavenumber and the angular frequency of the incident wave, respectively.

When a semi-infinite material is taken into consideration, the approximate solution of Equation (1) involving the second harmonic is given as Equation (2) [32]:

(2) $$u\left(x,t\right)=u^{0}+u^{1}=A\sin\left(kx-\omega t\right)-\frac{E\beta k^{2}A^{2}}{8\rho c^{2}}x\cos\left[2\left(kx-\omega t\right)\right]$$

where $E=\rho c^{2}$ is the Young’s modulus and c is the wave speed. Therefore, when a continuous plane wave is incident into the solid, the ultrasonic nonlinearity parameter β for the lossless material can be obtained by measuring the fundamental $A_{1}$ and second harmonic displacement $A_{2}$ after a propagation distance x. The resulted expression is thus derived as Equation (3):

(3) $$\beta =\frac{E_{2}}{E_{1}}=\frac{8}{xk^{2}}\left(\frac{A_{2}}{A_{1}^{2}}\right)$$

Equation (3) is widely used in nonlinear ultrasonic measurement to calculate the absolute ultrasonic nonlinearity. Once the amplitudes of fundamental $A_{1}$ and second harmonic $A_{2}$ are obtained, the nonlinearity parameter β can be calculated as a material property indicator.

On the other hand, various analytical expressions derived from the hyperelastic theory have been developed to account for the elastic contribution to the ultrasonic nonlinearity, which is related to the third-order elastic constants and is sensitive to the material microstructural variations. The well-known hyperelastic constitutive models, including the Neo-Hookean, Mooney-Rivlin, Yeoh, Ogden, and Murnaghan models [33,34], can describe the behaviors of nonlinear materials. Among them, Murnaghan’s model is a popular and classical hyperelastic model used to study the wave propagation in a quadratic nonlinear material. Combining the wave equation, the ultrasonic nonlinearity parameter can be represented by Murnaghan constants [33] as Equation (4):

(4) $$\beta =-\frac{3\left(\lambda +2\mu \right)+2\left(l+2m\right)}{\lambda +2\mu }$$

where  λ and μ are the Lame constants; l and m are the Murnaghan third-order elastic constants. With these elastic constants, the ultrasonic nonlinearity parameter can be calculated explicitly for numerical model verification.

## 3. Ultrasonic Scattering from Rough Surfaces

**Figure 1:**
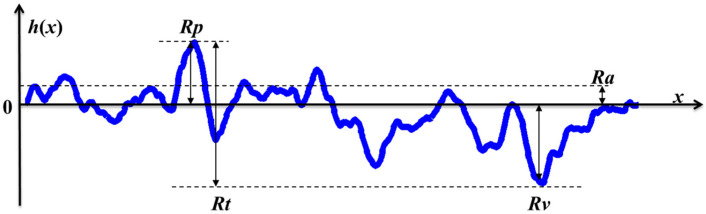
Schematic diagram of 1D rough surface and typical roughness parameters.

Generally, rough surfaces can be categorized into simplified regular surfaces, such as sinusoidal, triangular, rectangular, and stochastically dominated random surfaces. Different roughness parameters have been defined to characterize the surfaces. The peak $R_{p}$, valley $R_{v}$, average $R_{a}$, and total $R_{t}$ are defined to characterize the surface h(x), as shown in Figure 1. Moreover, commonly used root-mean-square (RMS) roughness $R_{q}$ are defined within a sampling length $L_{s}$ as Equation (5):

(5) $$R_{q}=\sqrt{\frac{1}{L_{s}}\int_{0}^{L_{s}}h^{2}\left(x\right)\cdot dx}$$

The general statistical characteristic of the rough surface h(x) can be expressed using the probability density function (PDF). For classic random cases, the central limit theorem implies that the PDF can be described as the Gaussian distribution, as expressed in Equation (6):

(6) $$p\left(h\right)=\frac{1}{R_{q}\sqrt{2\pi }}exp\left(-\frac{h^{2}}{2R_{q}{}^{2}}\right)$$

**Figure 2:**
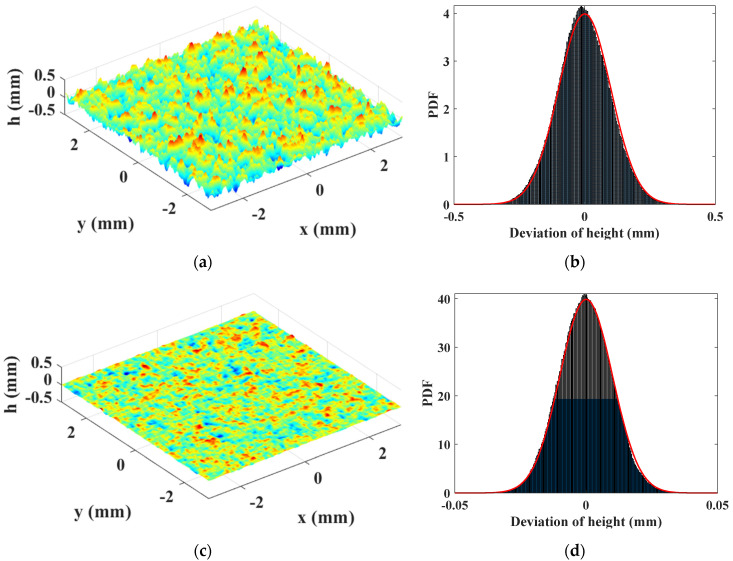
2D rough surface profiles with different parameters and their height distributions: (**a**) $L_{c}=0.1\;\mathrm{mm}$ and $R_{q}=0.1\;\mathrm{mm}$; (**b**) Probability density distribution of (**a**); (**c**) $L_{c}=0.1\;\mathrm{mm}$ and $R_{q}=0.01\;\mathrm{mm}$; (**d**) Probability density distribution of (**c**).

The PDF only deal with the amplitudes variation, so another function should be applied to represent the lateral variation. An autocorrelation function is defined for a Gaussian surface h(x,y) as Equation (7):

(7) $$C\left(\vec{r_{0}}\right)=\frac{\langle h\left(\vec{r}\right)h(\vec{r}+\vec{r_{0}})\rangle }{R_{q}{}^{2}}=exp\left[-\left(\frac{x^{2}}{L_{cx}^{2}}+\frac{y^{2}}{L_{cy}^{2}}\right)\right]$$

where $\vec{r_{0}}$ refers to the separation distance between two points; $\vec{r}$ indicates a radius vector from the origin. $L_{cx}$ and $L_{cy}$ refer to the correlation lengths in the x- and y-directions, defined as the distances where the correlation function falls to 1/e of its maximum value. For isotropic rough surfaces, $L_{cx}=L_{cy}=L_{c}$. By controlling the RMS value $R_{q}$ and the correlation length $L_{c}$, commonly used moving average method can be applied to generate random surfaces [13]. Examples of 2D random surfaces were generated with a Gaussian height density function and an exponential autocorrelation function, as shown in Figure 2. Clearly, the surface profile changes significantly with $R_{q}$ value. What should be mentioned is that the generated surface profiles with the same parameters are different from each process due to the random nature. Therefore, numerous studies should be conducted for the same rough surface to obtain a general rule with respect to the statistical parameters $R_{q}$ and $L_{c}$.

**Figure 3:**
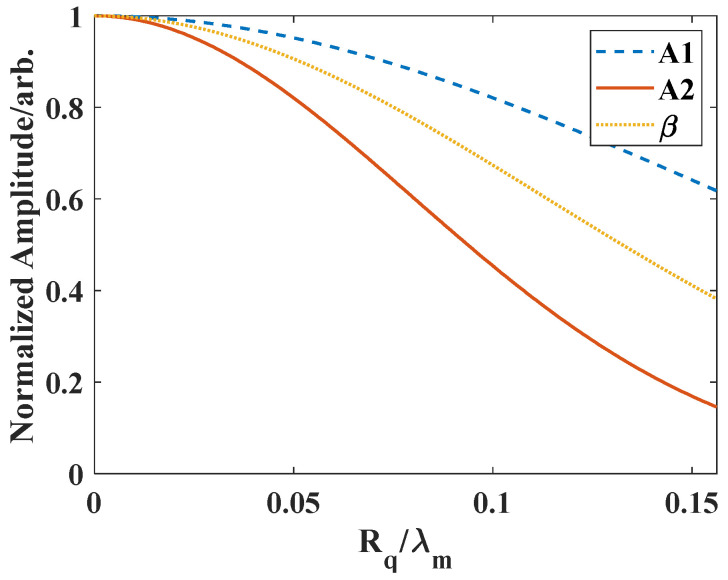
The change curves of normalized fundamental amplitude, second harmonic amplitude, and nonlinear parameter in the through-transmission mode with surface roughness $R_{q}$.

In ultrasonic nondestructive testing and monitoring, the rough surface may render the ultrasonic signals unpredictable due to the complicated wave scattering. When the slope of the surface is sufficiently small, i.e., $R_{q}\ll L_{c}$, The wave scattering from random rough surface can be simplified into phase changes as PSA theory to deal with the problems of reflection and transmission as reported by Nagy and Rose [6]. For a normally incident longitudinal wave, the roughness modified reflection coefficient and transmission coefficient can be expressed as Equations (8) and (9) [6]:

(8) $$R=R_{0}\exp\left(-2R_{q}^{2}k^{2}\right)$$

(9) $$T=T_{0}\exp\left(-\frac{1}{2}R_{q}^{2}{\left(k_{T}-k\right)}^{2}\right)$$

where $R_{0}$ and $T_{0}$ refer to the reflection and transmission coefficients from a smooth surface; k and $k_{T}$ indicate the wavenumber in the incident and transmitted media. When nonlinear effect is considered, scaling factors $\exp\left(-8R_{q}^{2}k^{2}\right)$ and $\exp\left(-2R_{q}^{2}{\left(k_{T}-k\right)}^{2}\right)$ should be applied to the amplitude of the second harmonic in the pulse-echo and through transmission modes. As the nonlinear parameter is calculated as $\beta =\frac{8}{xk^{2}}\left(\frac{A_{2}}{A_{1}^{2}}\right)$, a scaling factor  s should be considered for nonlinearity parameter calculation with rough surfaces. In the through-transmission mode with direct contact measurement, the factor $s_{T}$ can be expressed as $s_{T}=\exp\left(-{\left(2\pi \frac{R_{q}}{\lambda_{w}}\right)}^{2}\right)$. Therefore, the roughness-induced variation in conventional ultrasonic testing is much larger than that in nonlinear ultrasonic testing. Figure 3 shows the comparison of the roughness-induced change of the fundamental and second harmonic, and the nonlinearity parameter in the through-transmission mode. As expected, the second harmonic and nonlinearity are much affected by the surface roughness. Therefore, applying nonlinear ultrasonic testing has high potential to conduct surface roughness measurement with small RMS and to bridge the gap between conventional ultrasonic method and the optical methods.

## 4. FE Modelling for Nonlinear Ultrasound

### 4.1. Unit-Cell Model Setup

The FE methods are sufficiently powerful to calculate problems of linear and nonlinear elastic wave propagation with complicated boundaries [18,35]. In this section, the rough surfaces with Gaussian random profiles were taken into consideration to investigate the variation on roughness. Firstly, Murnaghan material model from solid mechanic module in a commercial FE software Comsol Multiphysics v5.4 (Burlington, MA, USA) was adopted to simulate the nonlinear wave propagation [33]. The specimen is Al-1200 and its material properties are shown in Table 1 [36].

**Figure 4:**
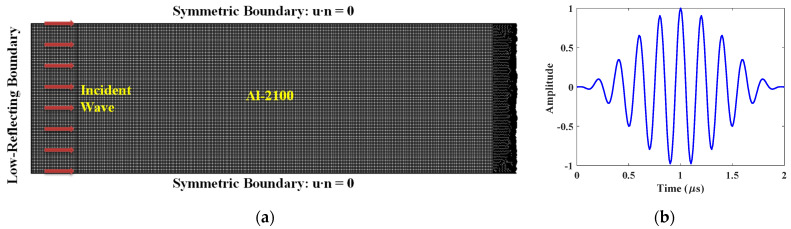
Schematic diagram of FE model: (**a**) geometry model and (**b**) incident waveform.

To avoid the wave scattering from the irrelevant boundaries, a large-size model with non-reflecting boundaries is usually applied [12,29,35]. However, the non-reflecting boundary may also bring in unpredictable numerical errors, making the received signals difficult to analyze. Unit-cell models, calculating only a represented domain, have been proposed and proven to be effective and efficient to simulate the wave propagation in infinite periodic boundaries [28,35]. As the correlation length in random surface describes periodicity of the surface, unit-cell model was applied in this study to facilitate a series of simulations for different rough surfaces. Moreover, although the 3D model is able to include the phenomena as much as possible, the 2D model with 1D surfaces has been proven to be effective to investigate the interaction between acoustic waves and rough surfaces [28,29,30]. These results have been well explained by the Kirchhoff approximation and validated by experimental results in previous literatures. In this study, a lot of simulations (50 realizations for each roughness) should be conducted for the rough surfaces. To complete all the simulation with proper accuracy at an acceptable computation cost, only 2D model is applied. Therefore, the schematic diagram of 2D FE model was shown in Figure 4a. A 10-cycle Gaussian-modulated sinusoidal wave with a central frequency of $f_{c}=5\;\mathrm{MHz}$ was applied as the incident wave to suppress the sidelobes. The incident waveform with a normalized amplitude is shown in Figure 4b. In order to generate an obvious nonlinear effect, the incident pressure of relatively high amplitude should be applied, and the peak amplitude of the pressure was set as 10 MPa. Symmetric boundaries were set for upper and lower boundaries. A symmetry condition is free in the plane and fixed in the out-of-plane direction, which is defined by the equation u·n=0. Besides, low-reflecting boundary was set at the left side to prevent the wave reflection.

As the FE methods numerically solve the partial differential equations by discretizing the problem domain into small elements, the careful choice of mesh size is critical to obtain accurate FE simulation results, especially for the nonlinear wave propagation problem. In order to investigate the variation as a function of the mesh, different sizes Δx of quadratic elements were set to mesh the domain and get the solutions. Element number per wavelength $N_{x}=\lambda_{w}/\Delta x$ was chosen as 1–30 to implement parametric study. Both the variations of $A_{1}$ and $A_{2}$ were investigated for nonlinear ultrasonic wave propagation. The amplitudes of $A_{1}$ and $A_{2}$ converge when the mesh is refined as $N_{x}=20$ [18]. As the longitudinal wavelength at 5 MHz is $\lambda_{w}=1.28\;\mathrm{mm}$, the element size was chosen as Δx=0.06 mm in this study. For the irregular domain near the rough surface, triangular element should be applied, and a further mesh refinement is required in order to ensure the convergence of the model [18]. The element size was set as 0.003 mm around the surface boundary, as shown in Figure 4a. Moreover, a generalized-α method was adopted as the time-dependent solver in this FE model to solve the transient wave propagation problem. To ensure the calculation convergence, the time increment should satisfy the Courant-Friedrichs-Lewy (CFL) condition, which is defined as $CFL_{m}=c\Delta t/\Delta x$, where Δt is the time step, Δx is the mesh size, and m is the element order. For quadratic elements, a value of 0.2 for $CFL_{2}$ has been demonstrated to be accurate for the final results [34]. Considering the finer mesh around the rough surface, the time increment Δt was chosen to be 1 ns in our model, which is also the sampling interval in the following data acquisition.

**Figure 5:**
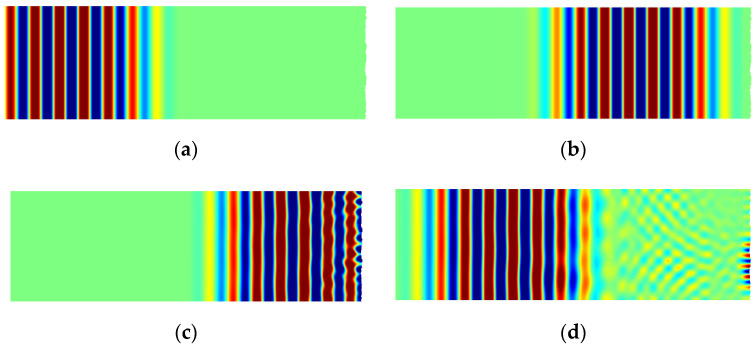
Snap shots at different times of wave propagation in the FE simulation: (**a**) t=1.5 μs; (**b**) t=3 μs; (**c**) t=4.5 μs; (**d**) t=6 μs.

To balance the computation amount and the accuracy, the width of model was set as 6 mm (≈5 λ). The correlation length was set as $L_{c}=0.1\;\mathrm{mm}\;(\approx \lambda_{w}/12)$ to ensure that the generated periodic surfaces have the same statistical characteristics. The RMS height $R_{q}$ varying from 0 to 200 μm $(\approx \lambda_{w}/6)$ were applied to investigate the influence of roughness on nonlinearity parameters. The random rough surfaces were generated by moving average method as described in Section 3. The total time was set as 9 μs to record both transmission and reflected signals. The typical results of simulated wave propagation at different times were shown in Figure 5. Besides the major energy of longitudinal wave reflecting from the rough surface, a small energy of shear wave can also be seen due to mode conversion at the surface.

### 4.2. Model Verification

**Figure 6:**
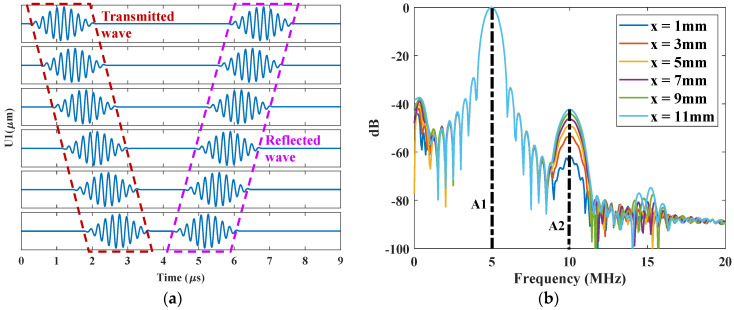
Received ultrasonic signals along the different propagation distances in the FE model: (**a**) A-scan signals and (**b**) the corresponding spectra of the transmitted parts.

To demonstrate the feasibility of this model for nonlinear ultrasonics, the nonlinearity parameter should be compared with the analytical results. Therefore, a smooth and flat surface was studied at first. When $R_{q}=0$, the horizontal displacement at the different positions were recorded, as shown in Figure 6a. Both transmitted and reflected signals were obtained and two components are separated when the receiving position is away from the surface. Otherwise, the transmitted and reflected waves will be overlapped, making it difficult to separate the corresponding higher harmonics. Therefore, only x=0~14 mm were applied for later analysis. The results of fast Fourier transform (FFT) of the transmitted waves are shown in Figure 6b. Higher harmonics, especially the second harmonic, are clearly generated in the spectrum. With the increase of propagation distance, the amplitude of the second harmonic $A_{2}$ increases, while the fundamental harmonic $A_{1}$ remains unchanged, as expected from Equation (2).

**Figure 7:**
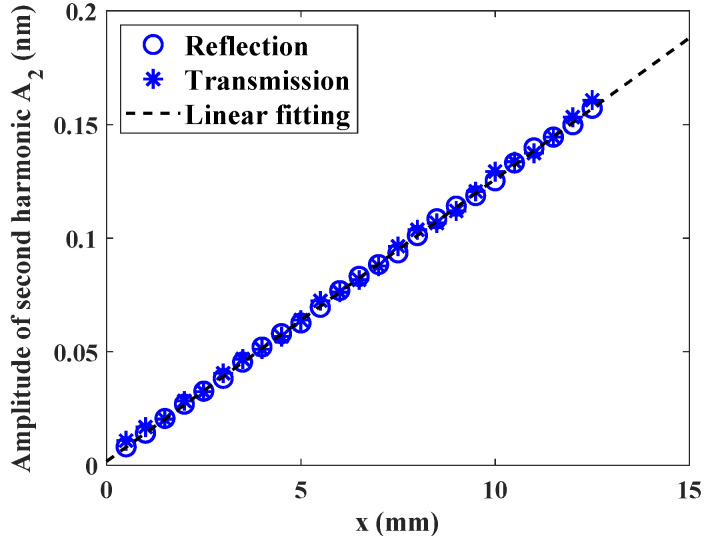
Amplitude variation of the second harmonic for reflected and transmitted waves with the propagation distance.

Both the absolute amplitude of second harmonic for reflected and transmitted waves were calculated for different propagation distance. The results are shown in Figure 7. For the transmitted wave, the amplitude of the second harmonic increases linearly with the propagation distance, which is the same as Equation (3). On the other hand, the amplitude of the reflected wave decreases with the propagation distance with the same change rate. When the reflected wave propagates back to the source position, the second harmonic decreases to zero. This similar phenomenon has been reported in [23,24]. To evaluate the accuracy of this model, the calculated nonlinearity parameter β is compared with the analytical result using Equation (3). The analytical value with Murnaghan constants is β=16.84. The calculated value using Equation (4) is β=17.48. Although there is little difference between both values (relative error is 3.8%) due to the errors from numerical calculation and the input signal with finite cycles, the nonlinearity parameters calculated from FE model and analytical solution are well agreed. Therefore, this FE model is accurate enough for modeling higher harmonic generation in a nonlinear elastic material and will be applied for rough surface evaluation.

## 5. Results and Discussion

### 5.1. Through-Transmission Mode

**Figure 8:**
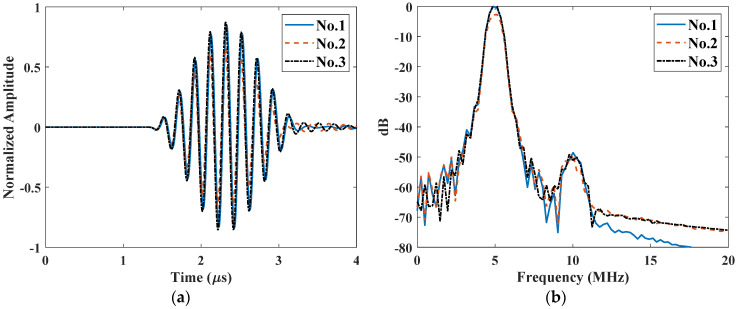
Three typical signals from the through-transmission model for the same rough surface ($R_{q}=50\;\mu m$): (**a**) A-scan waveforms and (**b**) their spectra.

To simulate the nonlinear ultrasonic testing in the through-transmission mode, a perfect coupled water film was added at the right boundary to mimic the couplant. To reduce the computation amount, the length of the solid material was reduced to 5 mm. The total time was set as 4 μs to record the transmission signals. Different rough surfaces were generated with RMS roughness $R_{q}$ ranging from 0 to 200 μm. A line average in the water layer was recorded as the transmitted signal and three typical signals for the same roughness $R_{q}=50\;\mu m$ as shown in Figure 8a. Compared to the smooth surface, a ring-down effect occurs for rough surfaces, making the received waveforms more complicated. Moreover, there are remarkable differences in the amplitude among the three signals due to the mutual interference between components of the signal transmitted through different parts of the rough surface. Figure 8b shows the corresponding spectra. Both fundamental and second harmonics can be clearly identified, while the amplitudes slightly vary among each realization. It demonstrates that rough surface will bring in some noises both in the linear and nonlinear ultrasonic measurement.

**Figure 9:**
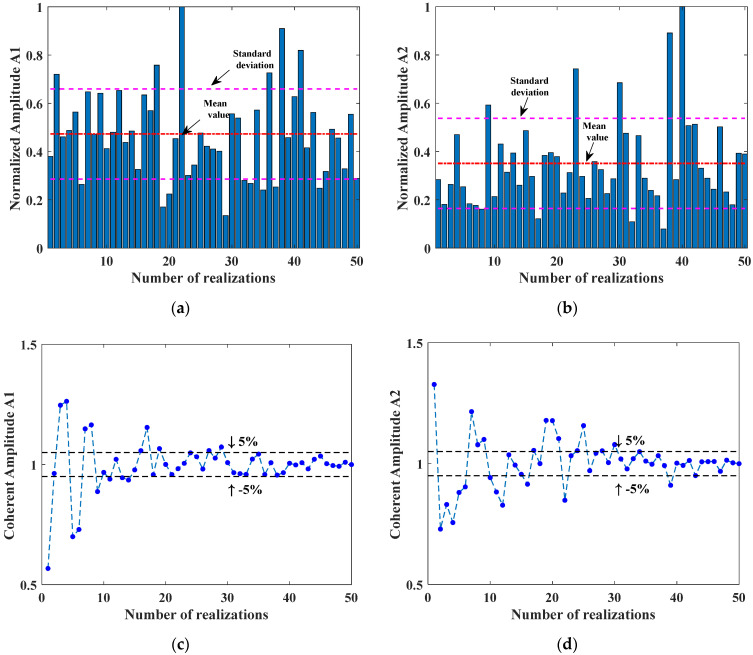
The influence of different surface realizations on the fundamental and second harmonic amplitudes for the same roughness ($R_{q}=200\;\mu m$). Variation of (**a**) A1 and (**b**) A2 for different realizations. The coherent amplitudes of (**c**) A1 and (**d**) A2 change with number of realizations.

In order to investigate the influence of surface profiles on the amplitude of transmitted wave, different realizations were conducted for the rough surface with the same roughness $R_{q}=200\;\mu m$. The normalized amplitudes of fundamental and second harmonics are shown in Figure 9a,b. Due to the random process of the rough surface realization, both amplitudes vary from each realization. For normalized fundamental amplitude, the arithmetic mean of the 50 realizations is 0.4730 and the standard deviation is 0.1867. For the second harmonic, the mean value and standard deviation are 0.3508 and 0.1866, respectively. Compared to the fundamental harmonic, the amplitudes of the second harmonic decrease much more. The fluctuations of both amplitudes are very large; therefore, sufficient realizations of rough surfaces for the same roughness are required to obtain the coherent signal, calculated as the average of multiple simulations. To fully remove the out-of-phase components from the scattered signals, a proper number of simulations should be determined to balance the accuracy and computation amount. The influence of the increasing number of realizations on the amplitude of coherent signal was investigated. Figure 9c,d show the variations of coherent fundamental and second harmonic wave for the roughness $R_{q}=200\;\mu m$, which is the maximum value in this study. When the number is few, the coherent amplitudes are not stable for both fundamental and second harmonics. When the number increases to more than 30, the coherent amplitudes of the fundamental wave are close to convergence, with a relatively small variation less than 5%. However, the coherent amplitudes of the second harmonic converge at a larger number around 50. The reason lies in that the second harmonic contains the information of the interaction between waves with smaller wavelength and rough surfaces. Therefore, to ensure the accuracy of the coherent wave for both fundamental and second harmonics, 50 simulations will be conducted repeatedly for each surface with the same roughness $R_{q}$ ranging from 0 μm to 200 μm.

**Figure 10:**
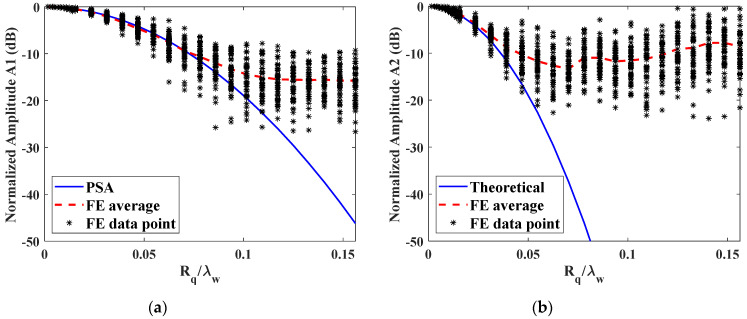
Variation of transmitted amplitudes from rough surfaces as a function of normalized roughness $R_{q}$ with respect to the smooth surface: (**a**) fundamental harmonic A1 and (**b**) second harmonic A2. The roughness is normalized by the incident wavelength $\lambda_{w}$. Results shown for FE simulations compared with PSA prediction.

The normalized amplitude variations as a function of the ratio of roughness to the incident wavelength are shown in Figure 10. When the roughness increases, both fundamental and second harmonic amplitudes vary for each realization. The average of 50 simulations was calculated as the coherent amplitude for each roughness to compare with the analytical solutions from the PSA theory. For the normalized fundamental amplitude, i.e., transmission coefficient in this case, the coherent amplitudes from FE simulation generally agree with the PSA results when the roughness is small. However, an obvious deviation appears when the roughness is larger than $0.07\;\lambda_{w}$ and increases further with the increasing roughness value $R_{q}$. It means that PSA fails to predict the transmission coefficient when the roughness is large, which is consistent with the result in the previous literature [2]. Therefore, as the roughness increases, Kirchhoff based analytical solution becomes increasingly inaccurate and a fully numerical approach is required.

For the second harmonic amplitude, as shown in Figure 10b, the FE results also show a generally decreasing trend with increasing roughness and starts to deviate from the PSA result at a smaller roughness value of around $0.03\;\lambda_{w}$. Moreover, the fluctuation of nonlinearity parameter is much larger than that of transmission coefficient due to the existence of the second harmonic, which is half the wavelength of the incident wave. Therefore, surface roughness will induce much more noises in the nonlinear ultrasonic measurement, which is the reason why careful surface treatment must be applied to the specimen prior to the measurement. Meanwhile, the second harmonic changes with the roughness at a higher rate than transmission coefficient, as predicted in Figure 3. When a roughness $R_{q}$ is as small as 20 μm corresponding to $1.56\%\;\lambda_{w}$, only a small change of 0.52 dB will be introduced to the transmission coefficient. Such subtle variation brings in difficulty to accurately measure this small roughness by conventional ultrasonic method. For the same roughness, the amplitude of second harmonic decreases to −1.70 dB. As the numerical results agree well with the PSA result when the roughness is smaller than $0.03\;\lambda_{w}$, PSA solution can be applied as the calibration curve for uncertainty analysis. Thus, when the second harmonic is applied for the measurement of nominal roughness for $R_{q}=20\;\mu m$, the standard uncertainty is calculated as $s_{Rq}=\sqrt{\frac{1}{n\left(n-1\right)}\overset{i=n}{\underset{i=1}{\sum }}{\left(R_{q,i}-\bar{R_{q}}\right)}^{2}}=0.45\;\mu m$ with n=50 observations. It indicates that the second harmonic generation is much more sensitive to the surface roughness. Therefore, nonlinear ultrasonic is very suitable to characterize the surface roughness, especially for the surfaces with small RMS.

### 5.2. Pulse-Echo Mode

**Figure 11:**
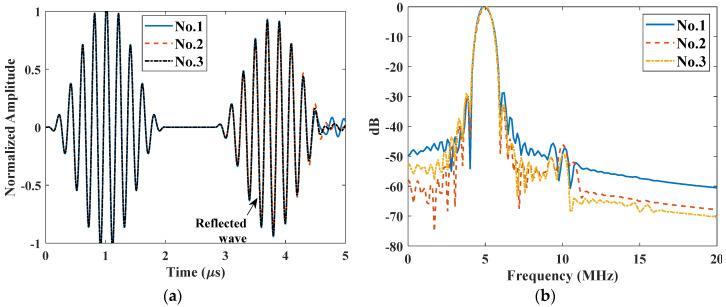
Three typical signals from the pulse-echo mode for the same rough surface ($R_{q}=50\;\mu m$): (**a**) A-scan waveforms and (**b**) the spectra of the reflected waves.

The nonlinear ultrasonic testing in the pulse-echo mode was also built and the model is the same as described in Section 4.1. A line average near the excitation position was recorded and three typical signals for the same roughness $R_{q}=50\;\mu m$ as shown in Figure 11a. Compared to the results from the smooth surface, a ring-down effect is also observed for rough surfaces. Similarly, there are obvious differences in the amplitude among the three realizations due to mutual interference from different parts of the rough surface. Both fundamental and second harmonics of the reflected wave vary from each realization, as shown in the corresponding spectra in Figure 11b. As illustrated in Figure 7, the second harmonic almost decreases to zero when the receiving position is close to the excitation. However, the second harmonics are clearly observed when it reflected from the rough surface. It means that the second harmonic can also be received with planar transducer rather than focused transducer or dual-element transducer as reported in the previous literatures [24,25,26,27]. As the accumulated second harmonic is zero for smooth surface, these remaining second harmonics only result from the roughness. Therefore, there is potential in measuring the surface roughness in the pulse-echo mode based on second harmonic generation.

**Figure 12:**
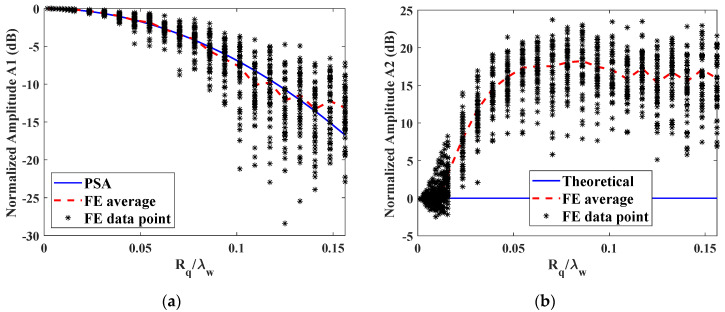
Variation of reflected amplitudes from rough surfaces as a function of normalized roughness $R_{q}$ with respect to the smooth surface: (**a**) fundamental amplitude A1 and (**b**) second harmonic amplitude A2. The roughness is normalized by the incident wavelength $\lambda_{w}$. Results shown for FE simulations compared with PSA prediction.

Similarly, rough surfaces with roughness $R_{q}$ ranging from 0 to 200 μm were investigated in the pulse-echo mode and 50 simulations were conducted repeatedly for each roughness. The normalized amplitude variations as a function of the ratio of roughness to the incident wavelength are shown in Figure 12. For the normalized fundamental amplitude, i.e., reflection coefficient in the pulse-echo mode, the coherent amplitudes from FE simulation generally agree with the PSA results when the roughness are smaller than $0.007\;\lambda_{w}$. However, an obvious deviation increases with the increasing roughness value $R_{q}$. Also, PSA fails to predict the reflection coefficient when the roughness is large. On the other hand, the second harmonic amplitude is close to zero when the roughness is small, which is like the reflection from smooth surface as shown in Figure 12b. When the roughness $R_{q}$ increases to 10 μm corresponding to $0.78\%\;\lambda_{w}$, the second harmonic amplitudes begin to increase quickly with the roughness. When the roughness is too large, the second harmonic amplitudes of reflected waves keep almost constant. When $R_{q}=70\;\mu m$, the averaged amplitudes increase to 17.33 dB. In contrast, the reflection coefficient decreases to −1.85 dB when $R_{q}=70\;\mu m$. It indicates that the second harmonic generation in the pulse-echo mode is very sensitive to measure the surface roughness within the range of $R_{q}=0.78\sim 5.47\%\;\lambda_{w}$.

Therefore, second harmonic generation can be applied to measure the surface roughness, not only in the through-transmission mode but also in the pulse-echo mode. Compared to the transmission coefficient or reflection coefficient, the second harmonic generation is more remarkably influenced by the rough surface. Therefore, surface roughness with relatively small RMS can be measured by nonlinear ultrasonics with a higher sensitivity. In the through-transmission mode, the surface roughness within the range of $R_{q}=0\sim 3\%\;\lambda_{w}$ can be measured. The effective range of surface roughness is $R_{q}=1\sim 5\%\;\lambda_{w}$ in the pulse-echo mode.

## 6. Conclusions

In this study, a numerical study has been carried out to investigate the surface roughness measurement based on nonlinear ultrasonic method. The Murnaghan hyperelastic material model was adopted in a unit-cell FE model to simulate higher harmonic generation in solids. The absolute value of the nonlinearity parameter was recovered, agreeing well with the analytical solution. Therefore, this FE model provides an effective way to analyze the higher harmonic generation in solids. Based on the verified FE model, the ultrasonic wave scattering with rough surfaces in both through-transmission and pulse-echo modes were investigated. Rough surfaces with Gaussian random profiles were generated by the moving average method and the RMS height $R_{q}$ varying from 0 to 200 μm $(\approx \lambda_{w}/6)$ were studied. 50 simulations were conducted repeatedly for each roughness to remove the out-of-phase components.

In the through-transmission mode, the transmission coefficient from FE simulation generally agrees with the PSA prediction when the roughness is smaller than $0.07\;\lambda_{w}$, while the second harmonic deviates at a smaller roughness value of around $0.03\;\lambda_{w}$. The higher decreasing rate of the second harmonic as a function of the roughness demonstrated that the nonlinear ultrasonic testing is very suitable to measure the surface roughness, especially for the surfaces with RMS as small as $0.03\;\lambda_{w}$. In the pulse-echo mode, the second harmonic is clearly observed when it is reflected from the rough surface with RMS larger than $0.007\;\lambda_{w}$ and its amplitude increases quickly with the roughness. As the accumulated second harmonic is zero reflected from smooth surface, the second harmonic generation in the pulse-echo mode is very sensitive to measure the surface roughness within the range of $R_{q}=0.78\sim 5.47\%\;\lambda_{w}$.

The numerical model introduced in this paper offers an efficient way to study the interaction of nonlinear ultrasonic wave with rough surfaces. The results can provide the correction terms for the absolute nonlinear ultrasonic measurement. The feasibility of the surface roughness with small RMS in the pulse-echo mode offers a potential early-stage monitoring method for the interfaces or inner surfaces of the in-service structures. In the future, work will be extended on a more realistic 3D model with 2D surface, and the experimental validation based on the dual-frequency ultrasonic transducer.

## Figures and Tables

**Table 1:** Material properties of Al-1200 [36].

Density	Lame Parameters		Murnaghan Third-Order Elastic Constants		
*ρ*	*λ*	*μ*	*l*	*m*	*n*
2737 kg/m^3^	57.0 GPa	27.6 GPa	−311 GPa	−401 GPa	−408 GPa

## Data Availability

The data presented in this study are available on request from the corresponding author.

## References

[1] Ma Z., Luo Z., Lin L., Krishnaswamy S., Lei M. (2019). Quantitative characterization of the interfacial roughness and thickness of inhomogeneous coatings based on ultrasonic reflection coefficient phase spectrum. NDT E Int..

[2] Ma Z., Lin L., Jin S., Lei M. (2019). Identification of the velocity, thickness, and interfacial roughness of coating using full time-domain urcps: Cross-correlation-based inverse problem. J. Nondestruct. Eval. Diagn. Progn. Eng. Syst..

[3] Benstock D., Cegla F., Stone M. (2014). The influence of surface roughness on ultrasonic thickness measurements. J. Acoust. Soc. Am..

[4] Ogilvy J.A. (1986). An estimate of the accuracy of the kirchhoff approximation in acoustic wave scattering from rough surfaces. J. Phys. D Appl. Phys..

[5] De Billy M., Quentin G. (1982). Backscattering of acoustic waves by randomly rough surfaces of elastic solids immersed in water. J. Acoust. Soc. Am..

[6] Nagy P.B., Adler L. (1987). Surface roughness induced attenuation of reflected and transmitted ultrasonic waves. J. Acoust. Soc. Am..

[7] Nagy P.B., Rose J.H. (1993). Surface roughness and the ultrasonic detection of subsurface scatterers. J. Appl. Phys..

[8] Lian M., Liu H., Zhou L., Zhang T., Liu B., Wang Y. (2019). Ultrasonic roughness measurement based on scattering attenuation. Surf. Topogr. Metrol. Prop..

[9] Shi F., Choi W., Lowe M.J.S., Skelton E.A., Craster R.V. (2015). The validity of kirchhoff theory for scattering of elastic waves from rough surfaces. Proc. R. Soc. A Math. Phys. Eng. Sci..

[10] Shi F., Lowe M.J.S., Xi X., Craster R.V. (2016). Diffuse scattered field of elastic waves from randomly rough surfaces using an analytical kirchhoff theory. J. Mech. Phys. Solids.

[11] Shi F., Lowe M., Craster R. (2017). Diffusely scattered and transmitted elastic waves by random rough solid-solid interfaces using an elastodynamic kirchhoff approximation. Phys. Rev. B.

[12] Shi F., Lowe M.J.S., Craster R.V. (2017). Recovery of correlation function of internal random rough surfaces from diffusely scattered elastic waves. J. Mech. Phys. Solids.

[13] Choi W., Shi F., Lowe M.J.S., Skelton E.A., Craster R.V., Daniels W.L. (2018). Rough surface reconstruction of real surfaces for numerical simulations of ultrasonic wave scattering. NDT E Int..

[14] Haslinger S.G., Lowe M.J.S., Huthwaite P., Craster R.V., Shi F. (2019). Appraising kirchhoff approximation theory for the scattering of elastic shear waves by randomly rough defects. J. Sound Vib..

[15] Liu M., Kim J.-Y., Jacobs L., Qu J. (2011). Experimental study of nonlinear rayleigh wave propagation in shot-peened aluminum plates—feasibility of measuring residual stress. NDT E Int..

[16] Matlack K.H., Kim J.Y., Jacobs L.J., Qu J. (2015). Review of second harmonic generation measurement techniques for material state determination in metals. J. Nondestruct. Eval..

[17] Yuan M., Lee T., Kang T., Zhang J., Song S.J., Kim H.J. (2015). Absolute measurement of ultrasonic non-linearity parameter at contact interface. Nondestruct. Test. Eval..

[18] Yuan M., Zhang J., Song S.-J., Kim H.-J. (2015). Numerical simulation of rayleigh wave interaction with surface closed cracks under external pressure. Wave Motion.

[19] Bovsunovsky A., Surace C. (2015). Non-linearities in the vibrations of elastic structures with a closing crack: A state of the art review. Mech. Syst. Signal Process..

[20] Na J.K., Yost W.T., Cantrell J.H., Kessel G.L. (2000). Effects of surface roughness and nonparallelism on the measurement of the acoustic nonlinearity parameter in steam turbine blades. Aip. Conf. Proc..

[21] Chakrapani S.K., Howard A., Barnard D. (2018). Influence of surface roughness on the measurement of acoustic nonlinearity parameter of solids using contact piezoelectric transducers. Ultrasonics.

[22] Kim J., Ha H.P., Kim K.M., Jhang K.Y. (2020). Analysis of the influence of surface roughness on measurement of ultrasonic nonlinearity parameter using contact-type transducer. Appl. Sci..

[23] Romer A., Kim J.Y., Qu J.M., Jacobs L.J. (2016). The second harmonic generation in reflection mode: An analytical, numerical and experimental study. J. Nondestruct. Eval..

[24] Best S.R., Croxford A.J., Neild S.A. (2014). Pulse-echo harmonic generation measurements for non-destructive evaluation. J. Nondestruct. Eval..

[25] Zhang S., Li X., Jeong H., Cho S., Hu H. (2017). Theoretical and experimental investigation of the pulse-echo nonlinearity acoustic sound fields of focused transducers. Appl. Acoust..

[26] Jeong H., Zhang S., Li X. (2018). Improvement of pulse-echo harmonic generation from a traction-free boundary through phase shift of a dual element transducer. Ultrasonics.

[27] Jeong H., Cho S., Shin H., Zhang S., Li X. (2020). Optimization and validation of dual element ultrasound transducers for improved pulse-echo measurements of material nonlinearity. IEEE Sens. J..

[28] Choi W., Skelton E., Lowe M.J.S., Craster R. (2013). Unit cell finite element modelling for ultrasonic scattering from periodic surfaces. AIP Conf. Proc..

[29] Wang Z., Cheng J. (2021). Numerical and analytical study for ultrasonic testing of internal delamination defects considering surface roughness. Ultrasonics.

[30] Zhang J., Drinkwater B.W., Wilcox P.D. (2012). Effect of roughness on imaging and sizing rough crack-like defects using ultrasonic arrays. IEEE Trans. Ultrason. Ferroelectr. Freq. Control..

[31] Li X., Fu Y., Zhang F., Rao Y. (2020). Detecting small flaws in two-phase ti-6al-4v with rough surfaces. Ultrasonics.

[32] Green R.E. (1973). Ultrasonic Investigation of Mechanical Properties.

[33] Murnaghan F.D. (1937). Finite deformations of an elastic solid. Am. J. Math..

[34] Rushchitsky J.J. (2009). Analysis of a quadratic nonlinear hyperelastic longitudinal plane wave. Int. Appl. Mech..

[35] Yuan M.D., Tse P.W., Xuan W.M., Xu W.J. (2021). Extraction of least-dispersive ultrasonic guided wave mode in rail track based on floquet-bloch theory. Shock Vib..

[36] Smith R.T., Stern R., Stephens R.W.B. (1966). Third—order elastic moduli of polycrystalline metals from ultrasonic velocity measurements. J. Acoust. Soc. Am..

